# The Passage of CARA: Implications for Patients With Cancer Pain

**DOI:** 10.6004/jadpro.2016.7.6.1

**Published:** 2016-09-01

**Authors:** Pamela Hallquist Viale

On July 22, 2016, President Barack Obama signed a bill into law that could have the potential to impact our practice. The Comprehensive Addiction and Recovery Act (CARA) is aimed at curbing the national opioid epidemic, a troubling phenomenon leading to increased deaths from drug overdoses. The Centers for Disease Control and Prevention (CDC) report that almost 500,000 people died of drug overdoses from 2000 through 2014 (CDC, 2016), clearly not what providers expect when writing opioid prescriptions for pain. There are compelling reasons for us to develop a means to combat opioid addiction and drug overdoses in an attempt to save lives.

However, pain is a feared and fearsome symptom often experienced by patients with cancer. In oncology, advanced practitioners (APs) have struggled for decades with developing and implementing optimal and comprehensive ways to assess and effectively manage the significant pain that can often be associated with this disease.

## WHAT DOES CARA MEAN?

The new law authorizes $181 million in funding for state programs to fight opioid addiction and includes the following components (American Society of Clinical Oncology [ASCO], 2016a):

(1) A requirement that the US Department of Health and Human Services (HHS) create a task force for the study of best practices for chronic and acute pain management

(2) A requirement to make naloxone more available and expand potential grants to states for the implementation of "standing orders" for the drug

(3) Reauthorization of the National All Schedules Prescription Electronic Reporting (NASPERS) Act and grants for state drug-monitoring programs

(4) Grants to states to combat the opioid epidemic and expansion of treatment for addiction

(5) "Partial fills" for opioids, giving patients less than the original prescription

Obviously, the recreational use of opioids is dramatically different from their use in cancer pain. Additionally, the use of opioids for chronic, non–cancer-related pain can be a difficult and complicated issue. However, it is reasonable for APs working in oncology to be wary of new legislation that could potentially affect the optimal pain management for our patients that we strive for.

This past May, ASCO released a policy statement on opioid therapy for patients with cancer. The policy focuses on this population and their unique needs, providing principles to address both the public health concerns and the mandate we have to provide appropriate pain management of our patients (ASCO, 2016b). A partial list of the pertinent facts noted in the ASCO statement reflects that:

(1) Patients with cancer are a special population and should be mostly exempt from restrictive regulations or limited doses of opioids

(2) Provider education is recommended and should be evidence-based and by specialty

(3) Prescription limits should not be enacted for patients with cancer if allowed to impact their access to medication

(4) Prescription drug-monitoring programs must consider the provider who is prescribing for cancer pain and the specialty, patient population, and additional factors that are appropriate in treating cancer pain

(5) Patient screening and assessment before and during opioid treatment should be determined by the treating physician

(6) Wider availability of naloxone is supported by ASCO

## PAIN AND SURVIVORSHIP

Judy Paice, PhD, RN, an AP and champion of cancer pain management in patients for many years, is the lead author of ASCO’s most recent guideline for the management of chronic pain in survivors of adult cancers. The guideline notes that with our incredible advancements in cancer diagnosis and treatment, more individuals are living with a history of cancer than ever before (Paice, Lacchetti, & Bruera, 2016). Although this is a laudable achievement, cancer survival can produce chronic pain, which can seriously affect patients and has been measured to be as high as 40% (Paice et al., 2016). Using an evidence-based approach, the authors note there is a "bottom line" to each component of the guideline, and provide a qualifying statement followed by recommendations for screening and comprehensive assessment; treatment and care options; nonpharmacologic interventions; pharmacologic interventions; use of opioids; and, importantly, risk assessment, mitigation, and universal precautions with opioid use.

## CAREFUL MONITORING

It is my hope that the passage of CARA will not have the undesired effect of impacting our ability to appropriately manage pain in our patients with cancer. It is true that the opioid epidemic is causing opioid-related deaths in record numbers and that at least half of all opioid overdose deaths implicate a prescription opioid. The management of non–cancer-related chronic pain is a complex issue and is not the focus of this editorial.

However, APs should stay informed on the repercussions made possible by the passage of CARA. We have wrestled for years with how to best manage cancer pain in our patients. We’ve celebrated the development of new agents and formulations of opioid medications, noting that pain management has become easier and more acceptable to patients when given in long-lasting preparations. We’ve educated ourselves on the optimal way to assess and address cancer pain in our unique population of patients. I would hate to see us backslide in our approach and ability to care for our patients’ pain—a serious and potentially debilitating symptom.

## References

[1] ASCO. (2016a). Opioid legislation expected to become law before summer congressional recess.. https://www.asco.org/advocacy-policy/asco-in-action/opioid-legislation-expected-become-law-summer-congressional-recess.

[2] ASCO. (2016b). ASCO releases principles for balancing appropriate patient access to prescription opioids with curbing misuse, abuse of these drugs.. Retrievedfromhttp://www.asco.org/advocacy-policy/asco-in-action/asco-releases-principles-balancing-appropriate-patient-access.

[3] Centers for Disease Control and Prevention. (2016). Increases in drug and opioid overdose deaths–United States, 2000-2014.. http://www.cdc.gov/mmwr/preview/mmwrhtml/mm6450a3.htm.

[4] Paice Judith A, Lacchetti Christina, Bruera Eduardo (2016). Management of Chronic Pain in Survivors of Adult Cancers: ASCO Clinical Practice Guideline Summary.. *Journal of oncology practice*.

